# Cyanobacteria Produce N-(*2-*Aminoethyl)Glycine, a Backbone for Peptide Nucleic Acids Which May Have Been the First Genetic Molecules for Life on Earth

**DOI:** 10.1371/journal.pone.0049043

**Published:** 2012-11-07

**Authors:** Sandra Anne Banack, James S. Metcalf, Liying Jiang, Derek Craighead, Leopold L. Ilag, Paul Alan Cox

**Affiliations:** 1 Institute for Ethnomedicine, Jackson, Wyoming, United States of America; 2 Department of Analytical Chemistry, Stockholm University, Stockholm, Sweden; 3 Craighead Beringia South, Kelly, Wyoming, United States of America; American University in Cairo, Egypt

## Abstract

Prior to the evolution of DNA-based organisms on earth over 3.5 billion years ago it is hypothesized that RNA was the primary genetic molecule. Before RNA-based organisms arose, peptide nucleic acids may have been used to transmit genetic information by the earliest forms of life on earth. We discovered that cyanobacteria produce N-(*2-*aminoethyl)glycine (AEG), a backbone for peptide nucleic acids. We detected AEG in axenic strains of cyanobacteria with an average concentration of 1 µg/g. We also detected AEG in environmental samples of cyanobacteria as both a free or weakly bound molecule and a tightly bound form released by acid hydrolysis, at concentrations ranging from not detected to 34 µg/g. The production of AEG by diverse taxa of cyanobacteria suggests that AEG may be a primitive feature which arose early in the evolution of life on earth.

## Introduction

**Figure 1:**
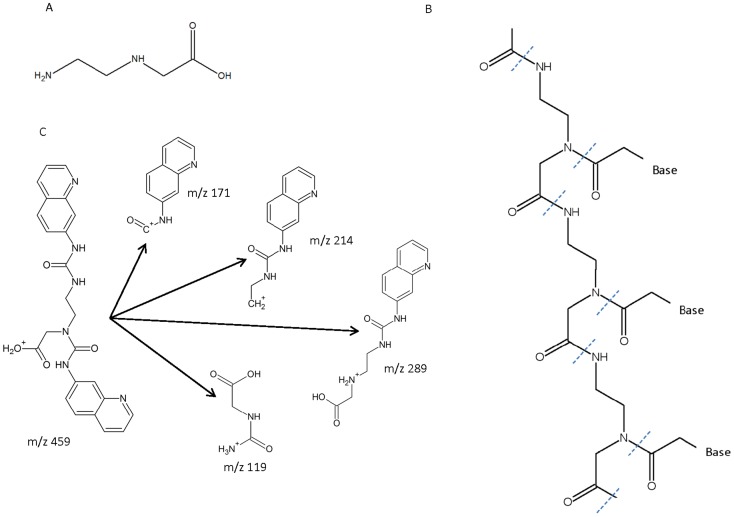
AEG [N-(*2*-aminoethyl)glycine] is a small molecule which when polymerized can form a peptide nucleic acid backbone. A, The AEG monomer. B, its proposed role as a peptide nucleic acid showing three AEG molecules each connected to a base by an acetyl linkage. C, Predicted fragmentation pattern of AQC derivatized AEG (*m/z* 459) following collision-induced dissociation to produce daughter ions of *m/z* 171, 214, 289 and 119. Predicted structures were produced using High Chem Mass Frontier 5.1 software (High Chem Ltd., Slovak Republic).

It has been hypothesized that RNA was the primary molecule for conveyance of genetic information by life on earth prior to the evolution of DNA over 3.5 billion years ago [1], [2]. However, the nature of primitive genetic systems before the evolution of RNA-based organisms is unclear. Polymers of N-(*2-*aminoethyl)glycine (AEG, Fig. 1), have been hypothesized as possible backbones of peptide nucleic acids (PNAs) that facilitated transmission of genetic information in the pre-RNA world [3], [4]. PNAs based on AEG have been synthesized and studied [5]–[9].

Cyanobacteria, photosynthetic Gram-negative bacteria, arose early in the earth’s history based on fossils from the Early Archean Apex Chert of Western Australia dating to 3.5 billion years ago [10]. Cyanobacteria were major contributors to the oxygenation of the earth’s atmosphere, and picoplankton such as *Prochlorococcus* and *Synechococcus* continue to play an important role in the global carbon balance [11]. Cyanobacteria are cosmopolitan, with some taxa occupying extreme habitats such as geothermal pools, hypersaline waters, or polar environments.

To determine if cyanobacteria produce AEG, we analyzed axenic cultures of cyanobacteria from the Pasteur Culture Collection (PCC) as well as environmental collections of cyanobacteria using triple quadrupole mass spectrometry (LC-MS/MS). Analyses were independently conducted at the Institute for Ethnomedicine in Jackson Hole, USA and at the Department of Analytical Chemistry of Stockholm University, Sweden.

## Results

**Figure 2:**
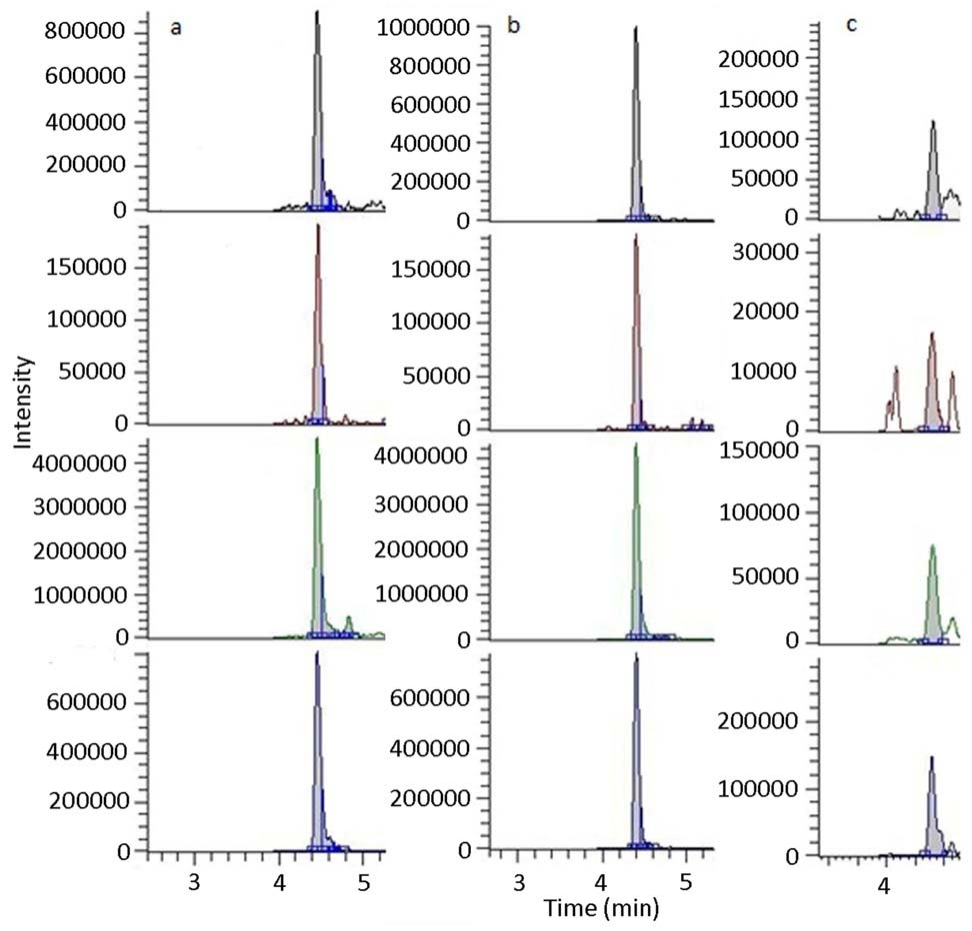
Cyanobacteria produce AEG [N-(*2*-aminoethyl)glycine], both in axenic PCC strains and environmental samples. Using triple quadrupole LC-MS/MS analysis, AEG was identified using a precursor ion *m/z* 459 and selective reaction monitoring of four transitions *m/z* 459 to *m/z* 289 (top pane), *m/z* 214 (second pane), *m/z* 171 (third pane), and *m/z* 119 (bottom pane). a, AEG was detected as a free or weakly bound compound in axenic *Nostoc* PCC 7120. b, A synthetic AEG standard. c, AEG in an extract of bound compounds from an environmental cyanobacterial sample collected at Benson Marina, Logan, Utah, USA.

We detected AEG as a free or weakly bound molecule in eight axenic PCC strains (Table 1; Fig. 2), which included both nitrogen-fixing and non-nitrogen fixing cyanobacteria from all five morphological cyanobacterial groups [12]. The total concentrations of free AEG and AEG liberated as a result of TCA extraction in the PCC strains ranged between 281 and 1717 ng/g. We also detected AEG in 15 different environmental samples of cyanobacteria we collected from diverse habitats around the world ranging from freshwater ponds in the deserts of Mongolia to marine samples from Qatar and river samples from Japan (Table 1; Fig. 2). The concentrations of AEG detected in environmental samples were generally higher than for the axenic samples, with concentrations ranging between not detected and 34 µg/g. After the removal of free and weakly bound AEG with a TCA extraction step, we hydrolyzed the precipitate, and detected AEG as a bound form in five environmental samples (Table 1). We could not detect AEG in blank BG11 media, before or after hydrolysis.

## Discussion

Included in our analysis were two strains (*Nostoc* PCC 7120 and *Synechocystis* PCC 6803) that have had their complete genomes sequenced [13], [14]. The production of AEG by both strains, which have only a 37% sequence similarity [14], suggests that the cyanobacterial ability to produce AEG is highly conserved. This, coupled with our discovery of AEG in all five morphological sections of cyanobacteria [12] suggests that the production of AEG may be a primitive feature within cyanobacteria. We are confident of our detection of AEG in cyanobacteria since LC-MS/MS is well-suited to the detection of small molecules in complex matrices [15] and has been shown to distinguish synthetic AEG from its structural isomers [16].

It is possible that AEG may occur in higher trophic levels in food chains based on cyanobacteria, since cyanobacteria are primary producers in many aquatic and some terrestrial ecosystems. The presence of AEG as a bound form in environmental samples suggests that biomagnification is possible as occurs for some of its isomers [17], [18].

The metabolic function of AEG in extant species of cyanobacteria and its potential evolutionary significance is unknown, although we do note that PNAs have been investigated by the pharmaceutical industry as possible gene silencers [19], [20]. Recent research shows synthetic xeno-nucleic acid to be susceptible to evolutionary change [21], [22]. It is tantalizing to hypothesize that the presence of AEG in cyanobacteria may be an echo of the pre-RNA world.

**Table 1:** Occurrence of N-(*2*-aminoethyl)glycine in axenic and environmental cyanobacterial samples.

Axenic Cyanobacterial Strain	Organism	Free AEG (ng/g)	Bound AEG (ng/g)	Habitatˆ	Geographic Locationˆ	Morphological Section
CC 6803	*Synechocystis* sp.	625	ND	FW	California, USA	section I
PCC 6712	*Chroococcidiopsis* sp.	1664	ND	FW	California, USA	section II
PCC 6506	*Oscillatoria* sp.	848	ND	unknown	unknown	section III
PCC 8106	*Lyngbya* sp.	281	ND	MW	North Sea, Germany	section III
PCC 7120	*Nostoc* sp.	1717	ND	unknown	unknown	section IV
PCC 73104	*Nodularia* sp.	644	ND	Terr	British Columbia, Canada	section IV
PCC 73105	*Anabaena* sp.	935	ND	FW	Cambridge, UK	section IV
PCC 7521	*Fischerella* sp.	1298	ND	Hot Spring	Yellowstone, USA	section V
Environmental Sample	Organisms	Free AEG (ng/g)	Bound AEG (ng/g)	Habitat	Geographic Location	Collection Date
Lake Houston	*Phormidium* sp.	10/60^a^	196/40^a^	FW	Texas, USA	Oct 2011
Cutler Marsh	*Phormidium* sp.	ND/ND^a^	2212/3240**^a^**	FW	Utah, USA	Sept 2011
Logan Pond	*Anabaenopsis* sp.	852^#^	10558	FW	Utah, USA	Sept 2011
Cutler Canyon River	*Phormidium* sp., Diatoms, Green Algae	ND/1450**^a^**	4061/540^a^	FW	Utah, USA	Sept 2011
Benson Marina	*Oscillatoria* sp., *Phormidium* sp.	ND	3285	FW	Utah, USA	Sept 2011
River- culture 1	Chroococcales	2090*		FW	Mie Prefecture, Japan	Feb 2010
River- culture 2	*Oscillatoria* sp.	811*		FW	Mie Prefecture, Japan	Feb 2010
River- culture 3	*Oscillatoria* sp.	1568*		FW	Mie Prefecture, Japan	Feb 2010
Altan Tevsh Spring	Oscillatoriales/Chroococcales/Green Algae/Diatoms	4780*		FW	Gobi Desert, Mongolia	Oct 2008
Tsagaan Tokhoi Spring	Oscillatoriales/Chroococcales/Green Algae/Diatoms	34318*		FW	Gobi Desert, Mongolia	Oct 2008
Khukh Ders Spring	Oscillatoriales/Green Algae	15004*		FW	Gobi Desert, Mongolia	Oct 2008
Mukhar Zadgai Spring	Oscillatoriales/Chroococcales/Green Algae	11604*		FW	Gobi Desert, Mongolia	Oct 2008
1 cultured cycad root endosymbiont +N0_3_^−2^	*Nostoc sp.*	2261*		Terr	Guam	Jun 2004
2 cultured cycad root endosymbiont +N0_3_^−2^	*Nostoc sp.*	6501*		Terr	Guam	Jun 2004
3 cultured cycad root endosymbiont +N0_3_^−2^	*Nostoc sp.*	6104*		Terr	Guam	Jun 2004
4 cultured cycad root endosymbiont -N0_3_^−2^	*Nostoc sp.*	9854*		Terr	Guam	Jun 2004
5 cultured cycad root endosymbiont -N0_3_^−2^	*Nostoc sp.*	7097*		Terr	Guam	Jun 2004
6 cultured cycad root endosymbiont -N0_3_^−2^	*Nostoc sp.*	2569*		Terr	Guam	Jun 2004
Coastal- culture	*Oscillatoria* sp.	3360*		MW	Mie Prefecture, Japan	Feb 2010
Inland Sea	*Lyngbya sp.*	3957” *		MW	Qatar	Apr 2011
Biscayne Bay	*Lyngbya sp.*	3152*/1110^a^		MW	Florida, USA	Jun 2009

ND =  not detected; FW =  fresh water; Terr =  terrestrial; MW =  marine water; # =  hydrolyzed TCA extract; * =  total AEG free + bound; ?Info from PCC; “Na0H hydrolysis; a =  quantification from Stockholm University; Morphological Sections from Ref 12.

## Materials and Methods

Axenic cyanobacterial strains were obtained from The Pasteur Culture Collection of Cyanobacteria, Paris (PCC) and grown for two months according to PCC recommendations. When sufficient biomass was attained, strains were lyophilized and free and weakly bound amino acids were extracted with TCA [23]. The remaining pellet was then hydrolyzed in 6 M HCl [23]. Extracts were derivatized with 6-aminoquinolyl-N-hydroxysuccinimidyl carbamate (AQC) and analyzed by LC-MS/MS [16]. Blank BG11 media containing nitrate was tested as a control. Environmental samples were similarly extracted and analyzed [16], [24] with the exception that several samples were hydrolyzed directly without TCA extraction, one sample was hydrolyzed in 6 M NaOH (Table 1), and the samples analyzed by Stockholm University were extracted using 10% TCA. The TCA extract was also hydrolyzed for all the Utah samples, one of which was positive for AEG (Table 1). Identification of AEG was based upon (a) the presence of the parent ion *m/z* 459; (b) retention time; (c) presence of product ions from collision-induced dissociation (*m/z* 171 quantifier ion; *m/z* 289, *m/z* 214, *m/z* 119 qualifier ions [*cf*. Ref 16 Fig. 1A]); and (d) ratios of qualifier ions relative to the quantifier ion. All samples were compared with an authenticated AEG standard (A1153 TCI America). Separation of AEG from its isomers β-N-methylamino-L-alanine (BMAA) and 2,4-diaminobutyric acid (2,4-DAB) was assessed using authenticated standards and culture extracts spiked with AEG and these standards, which showed a minimum separation of 0.15 min per isomer. The standard curve was prepared using six AEG concentrations (n ≥3) covering three orders of magnitude (7.4–740 nM AEG, r^2^ = 99.9%). The limits of detection (LOD) and the limits of quantification (LOQ) for AEG were 7.4 nM and 37 nM, respectively.
